# Early childhood lung function is a stronger predictor of adolescent lung function in cystic fibrosis than early *Pseudomonas aeruginosa *infection

**DOI:** 10.1371/journal.pone.0177215

**Published:** 2017-05-15

**Authors:** Jessica E. Pittman, Hannah Noah, Hollin E. Calloway, Stephanie D. Davis, Margaret W. Leigh, Mitchell Drumm, Scott D. Sagel, Frank J. Accurso, Michael R. Knowles, Marci K. Sontag

**Affiliations:** 1 Washington University School of Medicine, Division of Pediatric Allergy, Immunology, and Pulmonary Medicine, St. Louis, MO, United States of America; 2 University of North Carolina at Chapel Hill School of Medicine, Chapel Hill, NC, United States of America; 3 Stanford University School of Medicine, Department of Otolaryngology Head & Neck Surgery, Palo Alto, CA, United States of America; 4 Indiana University School of Medicine/Riley Hospital for Children, Section of Pediatric Pulmonology, Allergy, and Sleep Medicine, Indianapolis, IN, United States of America; 5 University of North Carolina at Chapel Hill, Department of Pediatrics, Chapel Hill, NC, United States of America; 6 University of North Carolina at Chapel Hill, Marisco Lung Institute, Chapel Hill, NC, United States of America; 7 Departments of Pediatrics and Genetics and Genome Sciences, Case Western Reserve University, Cleveland, OH, United States of America; 8 Department of Pediatrics, Children’s Hospital Colorado and University of Colorado School of Medicine, Aurora, CO, United States of America; University of Tübingen, GERMANY

## Abstract

**Objective:**

*Pseudomonas aeruginosa* has been suggested as a major determinant of poor pulmonary outcomes in cystic fibrosis (CF), although other factors play a role. Our objective was to investigate the association of early childhood *Pseudomonas* infection on differences in lung function in adolescence with CF.

**Methods:**

Two populations of subjects with CF were studied: from the Gene Modifier Study (GMS), 346 F508del homozygotes with severe vs. mild adolescent lung disease, and from the Colorado Newborn Screen Study (NBS) 172 subjects diagnosed with CF by newborn screening. Associations of *Pseudomonas* infection and lung function in early childhood with lung function in adolescence were investigated using multivariate linear regression analyses.

**Results:**

Among GMS subjects, those with severe adolescent lung disease had worse lung function in childhood (FEV_1_ 25 percentage points lower) compared to subjects with mild adolescent lung disease, regardless of early childhood *Pseudomonas* status. Among NBS subjects, those with lowest adolescent lung function had significantly lower early childhood lung function *and* faster rate of decline in FEV_1_ than subjects with highest adolescent lung function; early *Pseudomonas* infection was not associated with rate of FEV_1_ decline. The strongest predictor of adolescent lung function was early childhood lung function. Subjects with a higher *percentage* of cultures positive for *Pseudomonas* before age 6 or a lower BMI at 2–4 years old also had lower adolescent lung function, though these associations were not as strong as with early childhood lung function.

**Conclusions:**

In separate analyses of two distinct populations of subjects with CF, we found a strong correlation between lower lung function in early childhood and adolescence, regardless of early childhood *Pseudomonas* status. Factors in addition to early *Pseudomonas* infection have a strong impact on lung function in early childhood in CF. Further exploration may identify novel underlying genetic or environmental factors that predispose children with CF to early loss of lung function.

## Introduction

Cystic Fibrosis (CF) is a chronic, life-limiting genetic illness in which dysfunction of the CF transmembrane conductance regulator (CFTR) causes impaired mucociliary clearance, leading to chronic pulmonary disease, among other symptoms[1–4]. Lung disease in CF begins in infancy and is characterized by a cycle of chronic inflammation, infection, and airway damage causing progressive obstructive airways disease and loss of lung function[1–11]. Despite the early onset of pathology, patients with CF show marked variability in phenotype, disease severity, and survival[12–16].

Previous studies have tried to explain this heterogeneity by investigating factors associated with lower lung function in CF, including environmental (lower socioeconomic status, tobacco exposure)[17–20], nutritional[15, 21, 22], infectious (bacterial, viral, and fungal)[15, 21–32], and genetic (*CFTR* genotype and effects of various modifier genes)[12, 33–35]. *Pseudomonas aeruginosa* infection, in particular, has been associated with more rapid decline in lung function and more severe lung disease; earlier acquisition of *Pa* has been associated with poorer lung function in adulthood and higher risk of death in childhood. This has led to widespread adoption of *Pa* eradication protocols in children with CF[36, 37].

We have previously shown in a large, retrospective case-control study that earlier age of *Pa* infection (before 5 years of age) was strongly associated with severe (vs. mild) CF lung disease in adolescence and adulthood[29]. However, we hypothesized that multiple other factors are involved in determining lung function in early childhood in CF, and that early *Pa* infection alone would not account for differences in lung function in early childhood that persist into adolescence, even in a cohort of children diagnosed by newborn screening with subsequent aggressive early management of CF lung disease. Our primary objective was to investigate the association between early childhood lung function, early *Pa* infection, and adolescent lung function in subjects with CF. We also aimed to investigate other factors associated with early lung function in children with CF. To accomplish this, we tested our hypothesis in two separate datasets: the Gene Modifier Study (GMS), a retrospective, case-control study with annualized data, used in our original publication[29], and the Colorado Newborn Screen (NBS) Study, a prospectively collected, encounter-based study.

## Materials and methods

### Populations & variables

#### Gene Modifier Study (GMS)

A multicenter, retrospective case-control study of subjects with CF, all F508del homozygotes, classified as having either severe or mild adolescent lung function (defined as FEV_1_ in the highest or lowest quartiles for FEV_1_ percent predicted by birth cohort, respectively–an extremes of phenotype design), using annualized data from the CF Foundation Patient Registry (CFFPR)[29, 35]. Written informed consent was obtained for all subjects. This study was approved by the Institutional Review Board at the University of North Carolina, Chapel Hill. Analysis was restricted to subjects in our previous publication (n = 629)[29] with a) spirometry (PFTs) at 6–8 years of age, b) respiratory culture data before 6 years of age, and c) classification of adolescent lung disease severity at age 12 or older (to avoid overlap with early childhood FEV_1_ data).

Definitions of key variables:

- *Pa* status: ever/never recorded as having a culture positive for *Pa* before 6 years of age.
- Early childhood lung function: defined as the mean FEV_1_ percent predicted (FEV_1_) between 6–8 years of age (Wang)[38]. Best FEV_1_ between 6 and 8 years of age was also calculated. Calculations utilized all available spirometric data from the CFFPR.
- Mild vs. severe adolescent lung disease: defined based on GMS enrollment criteria[35].

#### Colorado Newborn Screen database (NBS)

The NBS database consists of prospectively collected, encounter-based clinical data (supplemented by CFFPR data) for subjects diagnosed with CF from 1982–2010 at the Children’s Hospital Colorado [39, 40]. Informed consent was obtained for all subjects. This study was approved by the Colorado Multiple Institutional Review Board, University of Colorado, Denver. For this study, analysis was restricted to subjects who a) were pancreatic insufficient, b) had BMI data available at 2–4 years of age, c) had respiratory culture data available before 6 years of age, d) had PFT data between 6 and 8 years of age, and e) had PFT data available at age 10 or older.

Definitions of key variables:

- Early childhood lung function: defined as the mean FEV_1_ percent predicted (FEV_1_) at 6–8 years of age (Wang)[38]. Best FEV_1_ at 6 to 8 years of age was also calculated. Calculations utilized all available spirometric data from the CFFPR.
- Adolescent lung function: defined as the mean FEV_1_ percent predicted (Hankinson)[41] for the last 3 years of available PFT data beginning at 10 years of age or older. Subjects were divided into even quartiles by adolescent lung function for bivariate analysis. This variable was treated as continuous for regression analysis.
- Number of spirometric measures: counts defined separately for both early childhood and adolescent lung function variables.
- Mean age at PFTs: defined separately for early childhood and adolescent variables.
- Early childhood infection status:
  ○ *Pa* status: ever/never culture-positive for *Pa* before 6 years of age,
  ○ Percentage of cultures positive for *Pa* prior to 6 years of age,
  ○ *S*. *aureus* status: ever/never culture-positive for *S*. *aureus* before 6 years of age,
  ○ Percentage of cultures positive for *S*. *aureus* prior to 6 years of age,
  ○ Number of respiratory cultures before 6 years of age.
- Early childhood nutritional status:
  ○ Mean BMI Z-score from 2 to 4 years of age,
  ○ Mean height Z-score from 2 to 4 years of age,
  ○ Mean weight Z-score from 2 to 4 years of age[42].
- Absolute change in FEV_1_ percent predicted from early childhood to adolescence: defined as mean early childhood FEV_1_ percent predicted minus the mean adolescent FEV_1_ percent predicted;
- Rate of change in FEV_1_ (percent predicted/year): defined as the absolute change in FEV_1_ percent predicted / (mean age at adolescent PFTs–mean age at early childhood PFTs)

Other variables included for both analyses were gender, race (Caucasian vs. non-Caucasian), ethnicity (Hispanic vs. non-Hispanic), year of birth, age at diagnosis, means of diagnosis (newborn screen, meconium ileus at presentation, and symptoms [respiratory, gastrointestinal, or failure to thrive]), *CFTR* genotype, and age of first recorded respiratory culture.

### Analyses (NBS, GMS)

Bivariate analyses used Student’s t-test and *χ*^2^ testing to compare means or proportions, respectively. Multiple comparisons were accounted for in the NBS cohort using ANOVA testing with Bonferroni correction. Comparisons consisted of:

1. Comparison of population characteristics by adolescent lung function group (GMS: severe vs. mild adolescent lung function; NBS: adolescent FEV_1_ quartile).
2. GMS: Comparison of early FEV_1_ in subjects with severe vs. mild adolescent FEV_1_, by early childhood *Pa* status.
3. GMS: Comparison of early FEV_1_ in subjects who were *Pa* positive vs. negative in early childhood, by severe vs. mild adolescent lung function group.
4. NBS: Comparison of early FEV_1_ by adolescent FEV_1_ quartile and by early childhood *Pa* status.
5. NBS: Comparison of early FEV_1_ in subjects *Pa* positive vs. negative in early childhood, by adolescent FEV_1_ quartile.
6. NBS: Comparison of rate of change and absolute change in FEV_1_ percent predicted from early childhood to adolescence by adolescent FEV_1_ quartile and early childhood *Pa* status.
7. NBS: Comparison of rate of change and absolute change in FEV_1_ percent predicted from early childhood to adolescence in subjects *Pa* positive vs. negative in early childhood by adolescent FEV_1_ quartile.

Multivariate linear regression with backwards elimination of covariates was used to define the association of early childhood characteristics and adolescent lung function (as the primary outcome) for the NBS dataset. A p value <0.05 was considered statistically significant for all bivariate analyses. Variables initially included in multivariate regression model were based on bivariate analyses and results from previous publications. Covariates included in the full model were: early childhood FEV_1_, gender, year of birth, diagnosis by newborn screen (yes/no), age at CF diagnosis, genotype (F508del homozygous vs. other), number of discrete PFTs included in early childhood FEV_1_ variable, number of discrete PFTs included in adolescent FEV_1_ variable, number of discrete cultures prior to 6 years of age, percentage of cultures positive for *Pa* prior to 6 years of age, percentage of cultures positive for *S*. *aureus* prior to 6 years of age, mean BMI percentile at 2 to 4 years of age, and number of discrete BMI values between 2 and 4 years of age. Variables were removed from the model based on evaluation of p values (p<0.05) and partial F testing.

## Results

Table 1 presents population characteristics for the GMS and NBS datasets (datasets available as supporting information files 1 & 2). NBS subjects were, on average, born later, diagnosed at a younger age (median 0.07 years vs. 0.35 years), and had respiratory culture data earlier than GMS subjects. Approximately 50% of NBS subjects were F508del homozygous, vs. 100% of GMS subjects. Approximately 17% (27/172) of the NBS cohort were diagnosed by meconium ileus and 4/172 were missed on the newborn screen and diagnosed later. The mean age at definition of adolescent lung function was 15.5 and 17.4 years for NBS and GMS subjects, respectively.

**Table 1:** Population characteristics of GMS and NBS subjects.

	GMS group	NBS group
	n = 346	n = 172
Female	49% (n = 169)	49% (n = 85)
F508del homozygous	100%	52% (n = 90)
Year of birth (median, range)	1985 (1979–1993)	1991 (1982–2000)
Age (years) at diagnosis (median)	0.35	0.07
Diagnosed by newborn screening	3% (n = 12)	81% (n = 140)*
Diagnosed with meconium ileus	25% (n = 88)	16% (n = 27)
***Culture data***		
Age (years) at first culture	2.5 (±1.9)	0.4 (±0.7)
Number of cultures prior to age 6	†	12.1 (±6.6)
*Pseudomonas aeruginosa* before age 6	63% (n = 218)	53% (n = 91)
Non-mucoid *Pa*	†	52% (n = 89)
Mucoid *Pa*	†	14% (n = 24)
Percentage of cultures *Pa* positive before age 6	†	11% (±17)
Age (years) first *Pa* under age 6 (of those *Pa* pos.)	3.5 (±1.8)	2.5 (±1.8)
*Staphylococcus aureus* before age 6	64% (n = 223)	84% (n = 144)
Methicillin-sensitive *S*. *aureus*	†	84% (n = 144)
Methicillin-resistant *S*. *aureus*	†	3% (n = 6)
Percentage of cultures *Staph* positive before age 6	†	32% (±25)
Age (years) first *Staph* under age 6	3.4 (±1.9)	1.7 (±1.6)
***Anthropometric data***		
Mean BMI Z-score at 2–4 years of age	†	-0.4 (±1.3)
***Lung function data***		
Mean FEV_1_ percent predicted age 6–8 years	86.8% (±20.4)	94.2% (±14.5)
Mean FEV_1_ percent predicted in adolescence	**	84.9% (±18.8)
Mean age of adolescent PFTs (or definition of GMS severity)	17.4 (±2.9)	15.5 (±3.6)

* Additional 5 subjects w/ false-negative newborn screening, remainder diagnosed with meconium ileus

† Limited data available (annualized CF Registry data)

** Adolescent lung function defined as severe vs. mild in GMS study by birth cohort

### GMS population

**Fig 1:**
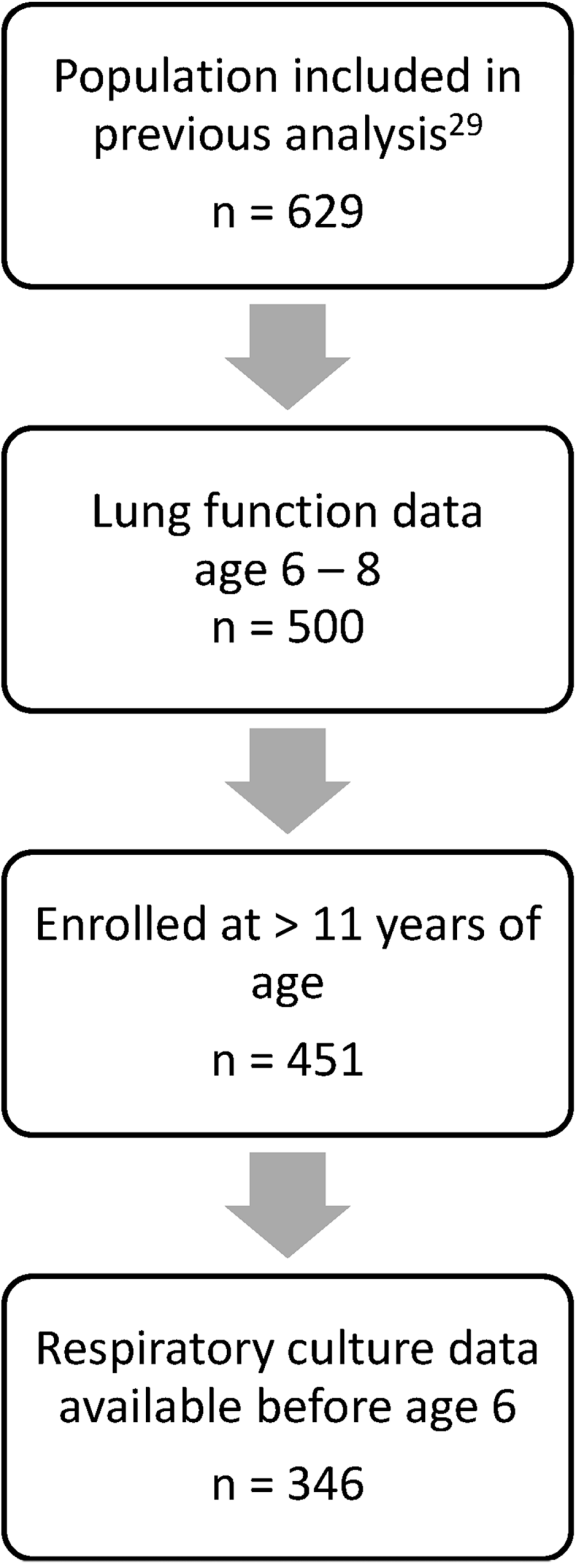
GMS population: Inclusion/Exclusion of subjects. Subjects from the parent GMS study included in current analysis were limited to those who a) had lung function data between age 6 and 8, b) were enrolled in the GMS study at 12 years of age or greater, and c) had respiratory culture data available prior to age 6. Definition of adolescent lung function occurred at time of GMS enrollment; age cutoff was created to separate time of definition of adolescent lung function from early childhood lung function.

Of the 629 F508del homozygous subjects initially enrolled and described elsewhere[29], 346 met inclusion criteria (Fig 1). Over 60% of subjects had at least one culture positive for *Pa* prior to 6 years of age.

GMS subjects were stratified into four subgroups by *Pa* infection status at age 6 (ever vs. never *Pa* positive prior to 6 years of age) and adolescent lung disease group (severe vs. mild). Subjects in the four sub-groups did not differ significantly by gender distribution, age at diagnosis, age of definition of adolescent lung function, or presence of other respiratory pathogens in early childhood (data not shown). Age of first culture was younger in subjects who were *Pa* positive in early childhood whether they had mild or severe lung function in adolescence (Mean age (years) at first culture in the mild adolescent lung disease group was 2.22 vs. 3.51 for *Pa* positive and negative subjects, respectively, p<0.05. Mean age at first culture in the severe adolescent lung disease groups were—2.18 vs. 3.51 for *Pa* positive and negative subjects, respectively, p<0.05). Similarly, *Pa* positive subjects had a higher mean number of respiratory cultures before age 6 regardless of lung function in adolescence (number of cultures among subjects with mild adolescent lung disease—4.56 vs. 3.11 for *Pa* positive and negative subjects, respectively, p<0.05. Number of cultures among subjects with severe adolescent lung disease—4.86 vs. 2.57 for *Pa* positive and negative subjects, p<0.05).

**Fig 2:**
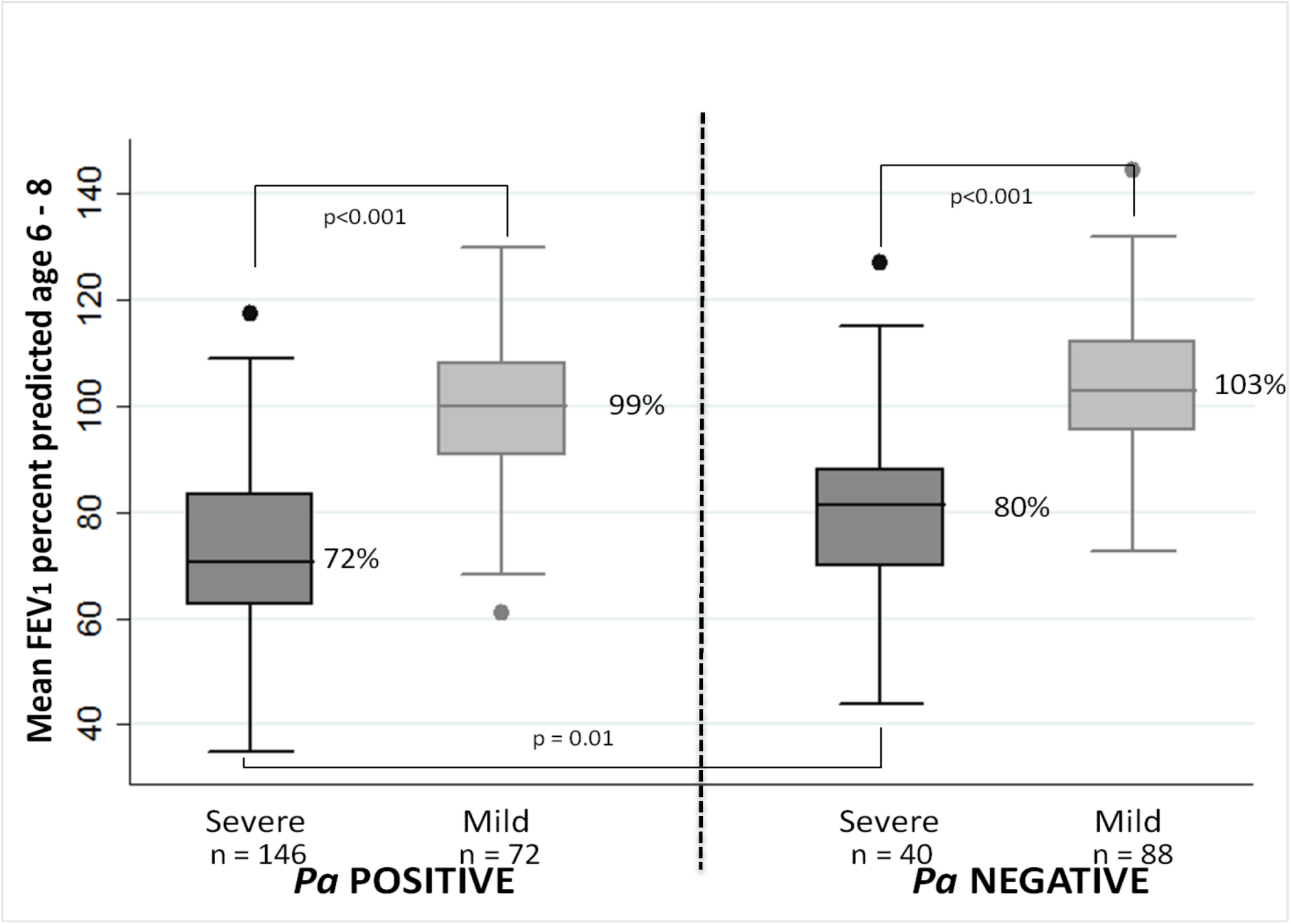
GMS population: Early childhood lung function by *Pa* status before age 6 and severe vs. mild lung disease in adolescence. Mean FEV_1_ percent predicted at 6 to 8 years of age is plotted by classification of lung disease in adolescence (severe vs. mild) and *Pa* infectious status up to age 6 (ever vs. never culture positive for *Pa*).

Fig 2 illustrates differences in early lung function among the four GMS subgroups. Among subjects who were *Pa* positive in early childhood, mean FEV_1_ at 6–8 years of age was lower in those with severe vs. mild adolescent lung disease (72.4% ± 15.8 and 99.4% ±13.1, respectively) (p<0.0001). Among *Pa-*negative subjects, there was a similar difference in early childhood FEV_1_ between those with severe vs. mild adolescent lung disease (79.1% ± 16.7 and 103.2% ± 14, respectively) (p<0.0001). In comparing subjects with severe lung disease later in life, mean early childhood FEV_1_ was ~8 percentage points lower in those subjects who were *Pa-*positive before age 6 (p = 0.01); however, there was no significant difference in early FEV_1_ between *Pa* positive and negative subjects with mild adolescent lung disease.

### NBS population

**Fig 3:**
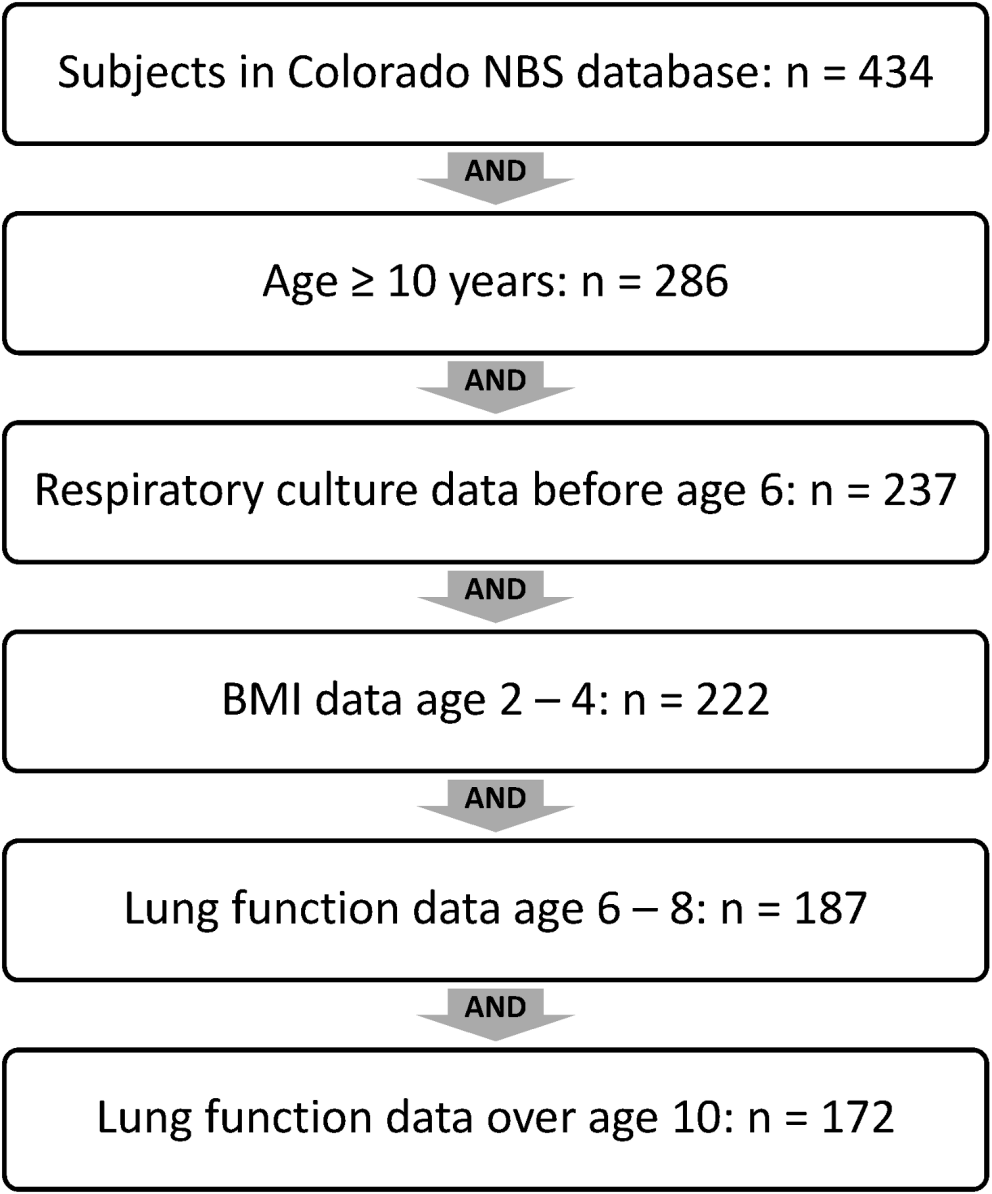
Colorado NBS population–Inclusion/Exclusion of subjects. Subjects in Colorado NBS analysis were limited to those 10 years of age or older at the time of analysis in order to have “adolescent” lung function data available. Subjects included in analysis were required to have data on respiratory cultures before age 6, BMI at age 2 to 4, lung function at age 6 to 8, and lung function over age 10.

Of 434 subjects included in the Colorado NBS database, 172 were included in final analysis; the majority excluded were not old enough to have “adolescent” lung function data available at 10 years of age or older (Fig 3).

#### Comparison by adolescent lung function quartile

NBS subjects were first divided into even quartiles by adolescent lung function (n = 43 in each quartile), then further divided by *Pa* status in early childhood. When comparing NBS subjects by lung function quartile in adolescence, subjects with lower adolescent lung function were born earlier and had a higher percentage of cultures positive for *Pa* prior to age 6 than adolescents with higher lung function (Table 2). There were no significant differences in nutritional status, age of first culture, number of cultures before age 6, percentage of subjects *Pa-*positive before age 6, or age at first *Pa*-positive culture between the adolescent lung function quartile groups. Subjects with lower adolescent lung function had significantly lower FEV_1_ at 6 to 8 years of age than those in the higher adolescent lung function quartiles (Table 2).

**Table 2:** Colorado NBS population characteristics by adolescent lung function quartile.

	Lowest quartile	2nd quartile	3rd quartile	Highest quartile	
	n = 43	n = 43	n = 43	n = 43	p (ANOVA or chi2)
Female	58%	42%	42%	56%	0.3
F508del homozygous	58%	44%	47%	60%	0.3
Year of birth	1989.6 (±5)	1991.8 (±4.6)	1992.2 (±3.8)	1993.5 (±5)	0.002
Age (years) at diagnosis	0.1 (±0.4)	0.1 (±0.1)	0.07 (±0.07)	0.1 (±0.2)	0.6
Diagnosed by newborn screening*	81%	72%	84%	88%	0.3
Diagnosed with meconium ileus	16%	21%	16%	9%	0.5
***Culture data***					
Age (years) at first culture	0.5 (±1)	0.3 (±0.3)	0.4 (±0.8)	0.2 (±0.3)	0.3
Number of cultures prior to age 6	11.3 (±7.6)	11.7 (±6.1)	11.3 (±5.7)	14.2 (±6.7)	0.1
*Pseudomonas aeruginosa* before age 6	67%	47%	49%	49%	0.2
non-mucoid *Pa*	65%	47%	47%	49%	0.2
mucoid *Pa*	19%	16%	14%	7%	0.4
Percentage of cultures *Pa* positive before age 6	31% (±26)	16% (±7)	18% (±12)	16% (±9)	0.0002
Age (years) first *Pa* under age 6	2.2 (±1.6)	2.7 (±2)	2.8 (±1.7)	2.3 (±2)	0.6
*Staphylococcus aureus* (all w MSSA) before age 6	79%	74%	88%	93%	0.08
MRSA	2%	2%	2%	7%	0.6
Percentage of cultures *Staph* positive before age 6	28% (±25)	32% (±27)	35% (±27)	33% (±21)	0.5
Age (years) first *Staph* under age 6	2 (±1.5)	1.7 (±1.5)	1.7 (±1.7)	1.5 (±1.6)	0.7
***Nutritional data***					
Mean BMI Z-score at 2–4 years of age	-0.8 (±2)	-0.3 (±0.9)	-0.3 (±1)	-0.1 (±0.8)	0.08
Mean BMI percentile at 2–4 years of age	36% (±28)	42% (±24)	40% (±27)	46% (±24)	0.37
number anthropomorphic measures age 2–4	5 (±3.8)	7 (±5)	6.9 (±3.4)	7.2 (±3.6)	0.049
***Lung function data***					
Mean FEV_1_ percent predicted age 6–8 years	84% (±13)	92.2% (±12.5)	95.5% (±11.2)	105.3% (±12.7)	<0.0001
Best FEV_1_ percent predicted age 6–8 years	96.2% (±15.3)	104.2% (±11.7)	106.1% (±11.8)	116.4% (±12.6)	<0.0001
Number of PFTs included age 6–8 years	7.5 (±5.7)	7.5 (±6.3)	6.6 (±3.3)	7.8 (±3.9)	0.7
Mean FEV_1_ percent predicted in adolescence	58.9% (±11.7)	82.1% (±3.9)	92.3% (±3)	106.4% (±7.2)	-
Best FEV_1_ percent predicted in adolescence	73.9% (±14.5)	95.6% (±8.6)	101.8% (±6.3)	113.7 (±8.5)	-
Number of PFTs included in adolescent data	21.9 (±18.8)	17.4 (±12.1)	11.7 (±11.6)	12.3 (±13.2)	0.003
Mean age of adolescent PFTs	16.9 (±3.6)	15.8 (±3.4)	15.2 (±2.4)	14.2 (±4.2)	0.004

* Additional 5 subjects w/ false-negative newborn screening, remainder diagnosed with meconium ileus

#### Comparison by adolescent lung function quartile and early *Pa* status

**Fig 4:**
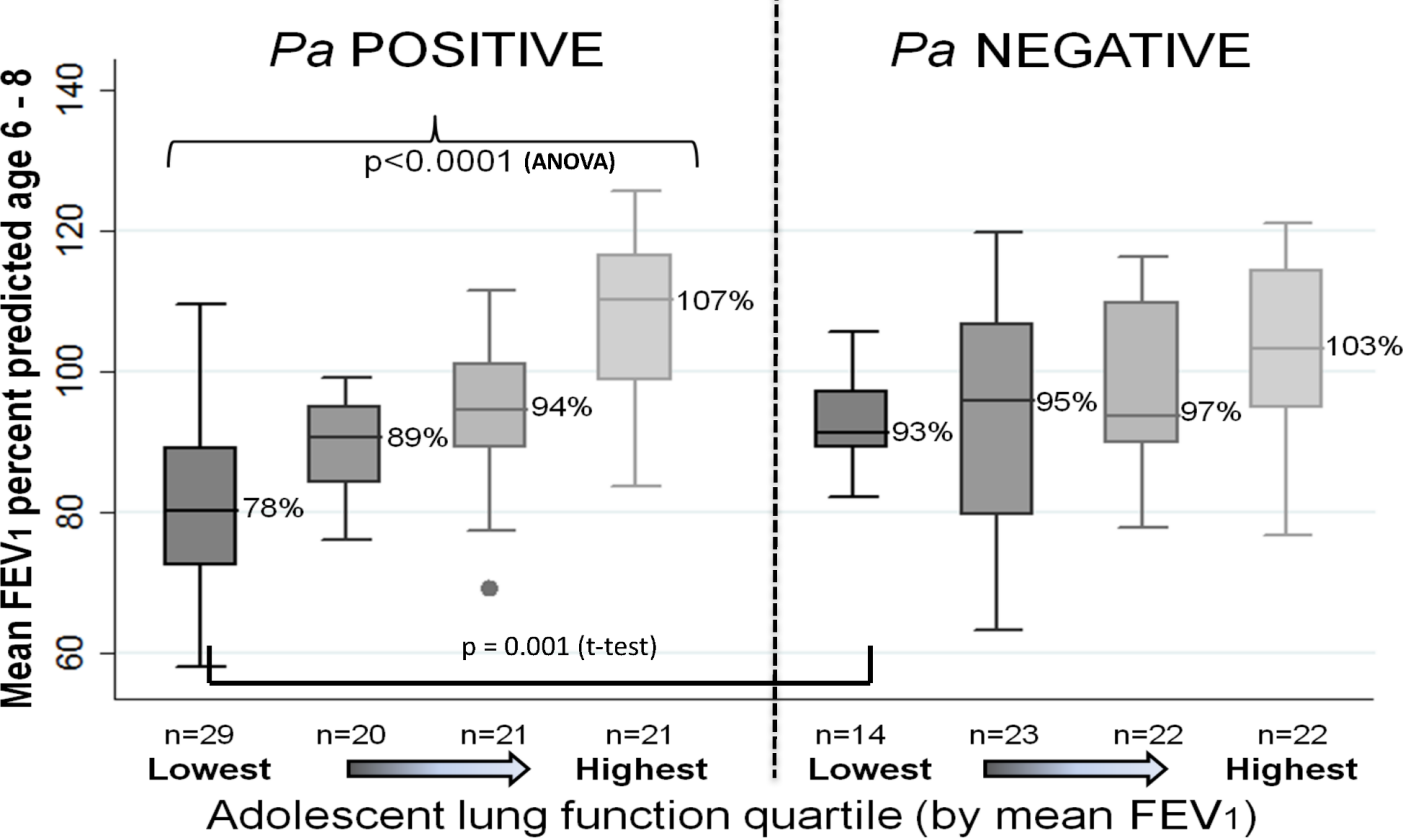
Colorado NBS Population: Early childhood lung function by adolescent lung function quartile and early childhood *Pa* status. Early childhood lung function (mean FEV_1_ at 6–8 years of age) by adolescent lung function quartile and early childhood *Pa* status (ever/never *Pa* positive before age 6). Subjects were first divided into even quartiles by adolescent lung function (n = 43 in each quartile), then further divided by *Pa* status in early childhood. Among *Pa* positive subjects, those in the lowest adolescent lung function quartile had significantly lower early childhood lung function compared to all other quartiles; differences in early childhood lung function among *Pa* negative subjects did not reach statistical significance. When comparing subjects within individual adolescent lung function quartiles, *Pa* positive subjects in the lowest adolescent lung function quartile had significantly lower early childhood lung function than *Pa* negative (p<0.001); otherwise there were no significant differences in early childhood lung function by *Pa* status within adolescent lung function quartiles.

Fig 4 illustrates differences in early childhood lung function by adolescent lung function quartile and early childhood *Pa* status. Early childhood FEV_1_ was significantly lower in subjects with poorer adolescent lung function who were *Pa* positive before age 6 (mean early childhood FEV_1_ 78% (±13), 89% (±7), 94% (±11), and 107% (±12) for lowest to highest adolescent lung function groups, p<0.0001). Though a similar pattern was seen in the *Pa* negative group, differences did not reach statistical significance (mean early childhood FEV_1_ 93% (±7), 95% (±16), 97% (±11), and 103% (±13) for lowest to highest adolescent lung function group, p = 0.065). When comparing *Pa* positive and negative subjects by adolescent lung function quartile, *Pa* positive subjects in the lowest adolescent lung function quartile had significantly lower early childhood lung function than *Pa* negative (78% (±13) vs. 93% (±6), p< 0.001); differences in early childhood FEV_1_ between *Pa* positive and negative subjects in the other quartiles were not statistically significant (p = 0.09, 0.18, 0.86 for 2^nd^ lowest, 3^rd^, and highest quartiles, respectively).

There was a significant decline in FEV_1_ percent predicted from early childhood to adolescence in the two lowest adolescent lung function quartiles, with a decrease of 25 percentage points and 10 percentage points in lowest and 2^nd^ lowest quartiles, respectively (p<0.0001 for both). There was no significant change in mean FEV_1_ from early childhood to adolescence in the higher two adolescent lung function quartiles (Table 3). Similarly, rate of decline in FEV_1_ from childhood to adolescence (percent predicted per year) was significantly higher in subjects in the two lowest adolescent lung function quartiles (Table 3).

**Table 3:** Colorado NBS Population: Absolute change and annual rate of change (percent predicted per year) in FEV_1_ percent predicted from early childhood to adolescence by adolescent lung function quartile. Absolute change in FEV_1_ was defined as mean FEV_1_ percent predicted_age 6–8_ minus mean FEV_1_ percent predicted_adolescent_. Annual rate of change was defined as absolute change in FEV/ (mean age adolescent PFTs–mean age early childhood PFTs).

	Mean FEV_1_		Best FEV_1_	
	mean	p	mean	p
**LOWEST QUARTILE**				
**change in % predicted (child—adolescent)**	-25.1	<0.0001	-22.3	<0.0001
**annual rate of change**	-2.8 (±2.1)	*†	-1.1 (±1.6)	*†
**2nd QUARTILE**				
**change in % predicted (child—adolescent)**	-10.1	<0.0001	-8.6	<0.0001
**annual rate of change**	-2.4 (±3.3)	‡	-1 (±1.5)	
**3rd QUARTILE**				
**change in % predicted (child—adolescent)**	-3.2	0.06	-4.4	0.03
**annual rate of change**	-1.7 (±2.1)		-0.5 (±2.1)	
**HIGHEST QUARTILE**				
**change in % predicted (child—adolescent)**	1.1	0.5	-2.7	0.09
**annual rate of change**	-1.1 (±2.7)		-0.5 (±1.8)	

* p value for difference among any of the 4 quartiles (ANOVA) < 0.001

† p value comparing lowest quartile to all other quartiles <0.001

‡ p value comparing 2nd quartile to highest quartile <0.003

Table 4 shows annual rate of decline (percent predicted per year) in FEV_1_ from early childhood to adolescence by adolescent lung function quartile and early childhood *Pa* status. Subjects with the lowest adolescent lung function had significantly faster annual rates decline in FEV_1_ than all other subjects; mean FEV_1_ percent predicted in the two highest quartiles appeared nearly stable from childhood to adolescence (by both percent predicted and rate of decline). Importantly, rate of decline did not differ significantly within adolescent lung function quartiles when comparing subjects by early childhood *Pa* status (Table 4).

**Table 4:** Colorado NBS Population: Annual rate of change in FEV_1_ percent predicted from childhood to adolescence by adolescent lung function quartile and *Pa* status before 6 years of age.

	**Adolescent Lung Function Quartile**							
	Lowest quartile n = 43		2nd quartile n = 43		3rd quartile n = 43		Highest quartile n = 43	
Mean (SD)	*Pa* negative n = 14	*Pa* positive n = 29	*Pa* negative n = 23	*Pa* positive n = 20	*Pa* negative n = 22	*Pa* positive n = 21	*Pa* negative n = 22	*Pa* positive n = 21
FEV_1_ age 6–8	92.9 (±6.8)*	79.7 (±13.2)	94.6 (±15.6)	89.5 (±7.1)^†^	97.1 (±11.5)	93.9 (±11)^†^**	103.2 (±13)	107.4 (±12.4)^†^**
Annual rate of change in FEV_1_% predicted	-2.6 (±0.9)	-2.8 (±2.5)	-1.2 (±1.9)	-0.9 (±1.1)	-0.4 (±1.6)^†^	-0.1 (±1.4)^†^	0.6 (±1.5)^†^	-0.1 (±1.7)^†^

* p<0.05 comparing *Pa* positive and negative groups within adolescent lung function quartile

† p<0.05 compared to lowest adolescent lung function quartile group with same *Pa* status

** p<0.05 compared to 2^nd^ adolescent lung function quartile group with same *Pa* status

For all comparisons discussed above, similar patterns were seen when comparing best early childhood FEV_1_ as opposed to mean (data not shown).

#### Linear regression

**Fig 5:**
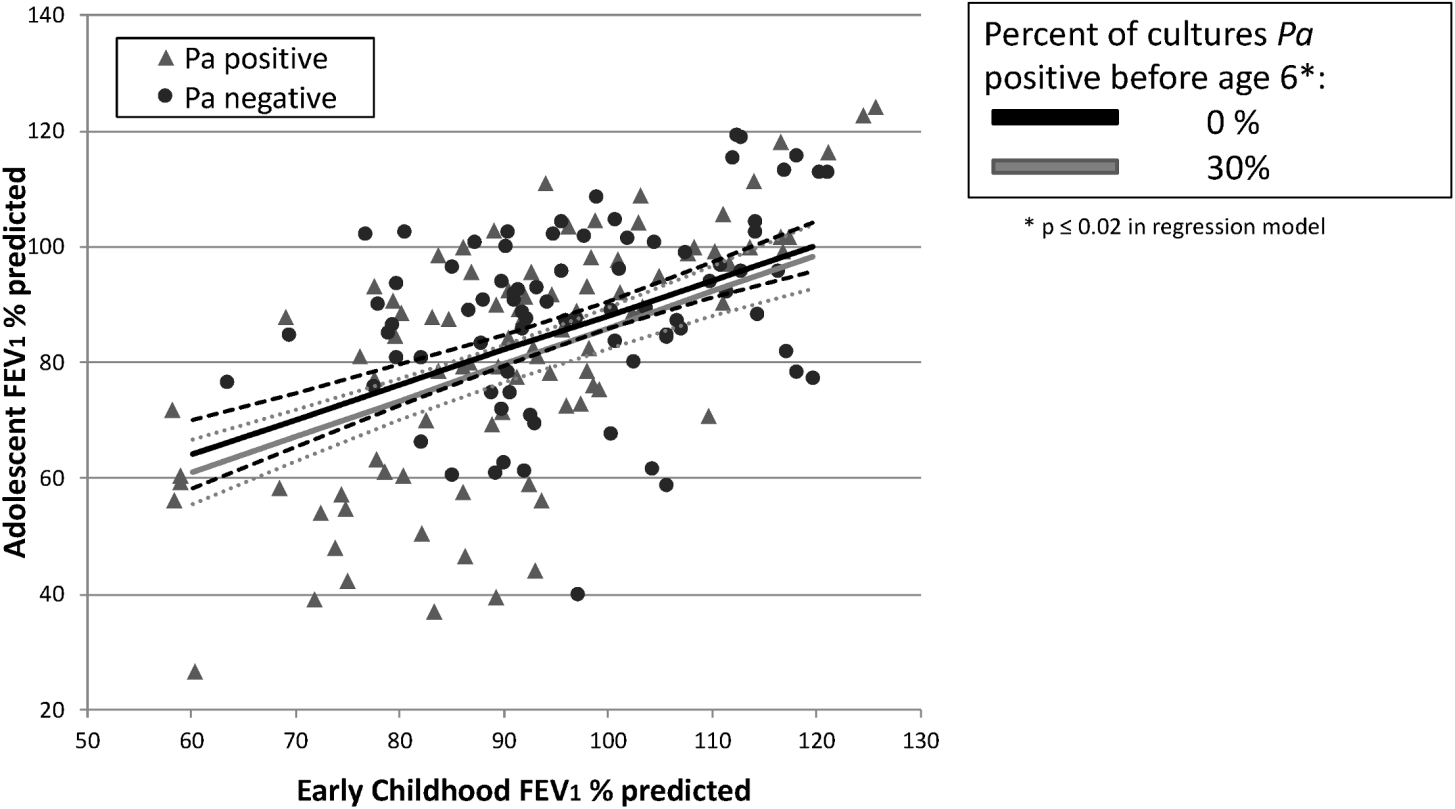
NBS linear regression: Predicted adolescent lung function is more strongly associated with early childhood FEV_1_ than with percentage of cultures positive for *Pa* in early childhood. Subject values for mean early childhood and adolescent FEV_1_ are shown in scatterplot, with subjects *Pa* positive in early childhood represented by gray triangles, *Pa* negative by black circles. Superimposed lines represent NBS linear regression results showing predicted adolescent FEV_1_ (with 95% confidence intervals—dashed lines) by early childhood FEV_1_. The black line shows predicted adolescent FEV_1_ by early childhood FEV_1_ when percentage of cultures positive for *Pa* prior to age 6 is held at 0% (25^th^ percentile); the grey line represents the predicted adolescent FEV_1_ when percentage of cultures positive for *Pa* prior to age 6 is held at 30% (approximately the 90^th^ percentile for our cohort). These values were all adjusted for gender, year of birth, age at diagnosis, number of cultures before age 6, BMI Z score at 2 to 4 years of age, and number of PFT tests included in early childhood and adolescent values (all held at mean). Predicted mean adolescent FEV_1_ increased by 19.7 percent predicted when early childhood FEV_1_ was increased from the 25^th^ percentile (85.5% predicted) to the 95^th^ percentile (117.4%), while it decreased by only 4.8% when percentage of cultures positive for *Pa* was increased from the 25^th^ percentile (0%) to the 92^nd^ percentile (30% positive), or 5.8% when increased to 36.8% positive (95^th^ percentile). Contribution to the explained variance of the full model was 0.1957 for mean early childhood FEV_1_ vs. 0.0157 for percentage of cultures positive for *Pa* before age 6.

Variables included in the final linear regression model (after backwards elimination of covariates) were: adolescent FEV_1_, early childhood FEV_1_, BMI Z-score at 2–4 years of age, percentage of cultures positive for *Pa* before age 6, number of cultures obtained before age 6, number of PFTs included in the early childhood lung function variable, number of PFTs included in the adolescent lung function variable, gender, year of birth, and age at diagnosis. We found a strong association between lower early childhood FEV_1_ and lower adolescent FEV_1_ (p<0.001, Table 5). Higher percentage of cultures positive for *Pa* prior to age 6 was also significantly associated with lower adolescent FEV_1_ (p = 0.02), though the effect appeared markedly less than that of early childhood FEV_1_. Contribution to the explained variance of the full model was 0.1957 for mean early childhood FEV_1_ vs. 0.0157 for percentage of cultures positive for *Pa* before age 6. In this model, predicted mean adolescent FEV_1_ increased by 19.7 percent predicted when early childhood FEV_1_ was increased from the 25^th^ percentile (85.5% predicted) to the 95^th^ percentile (117.4%), while it decreased by only 5.8% when percentage of cultures positive for *Pa* was increased from the 25^th^ percentile (0%) to the 95^th^ percentile (36.8% positive) (see Fig 5). Lower BMI in early childhood also showed a significant association with lower adolescent lung function (p = 0.005). Neither the simple presence of *Pa* prior to age 6 (yes/no dichotomous variable for at least one positive culture) nor the presence or percentage of cultures positive for *S*. *aureus* prior to age 6 were significantly associated with adolescent FEV_1_.

**Table 5:** Colorado NBS population: Linear regression, association with mean adolescent FEV_1_ percent predicted. Results of linear regression investigating association of early childhood events/exposures with mean adolescent FEV_1_ percent predicted. All variables with p<0.05 are shown; the model was also adjusted for gender, age at diagnosis, number of respiratory cultures prior to age 6, and number of PFTs used to calculate early childhood FEV_1_. Variables eliminated from the model included genotype (F508del homozygous yes/no), diagnosis by newborn screening, percentage of cultures positive for *S*. *aureus* prior to age 6, and number of measures included in BMI value.

			95% CI		
VARIABLE	ß coeff	SE	Lower 95%	Upper 95%	p value
Mean FEV_1_%predicted age 6 to 8	0.616	0.077	0.465	0.768	<0.001
Percent of cultures *Pa* positive before age 6	-0.159	0.070	-0.297	-0.021	0.024
Year of birth	1.03	0.319	0.397	1.66	0.002
BMI z-score age 2 to 4	2.37	0.826	0.743	4.01	0.005
Number of PFTs used to calculate mean adolescent FEV_1_	-0.324	0.078	-0.478	-0.17	<0.001

Also adjusted for gender, age at diagnosis, number of cultures age 0–6, number of PFTs age 6–8 (these variables were also included in regression analysis, but without significant p value).

## Discussion

We have shown in two separate, distinct cohorts of CF subjects that lower FEV_1_ in early childhood (6 to 8 years of age) is strongly associated with lower FEV_1_ in adolescence, and that this association is only partially explained by infection with *Pa* before age 6; thus, other genetic and/or environmental factors, likely in early childhood, must be playing a major role. Importantly, we have also shown a striking divergence in loss of lung function among CF patients, as subjects with worse lung function in early childhood had a significantly faster rate of FEV_1_ decline from childhood to adolescence compared to those with better early childhood lung function. This finding holds even for those diagnosed in infancy, was the majority of NBS subjects presented for management and treatment prior to the development of respiratory symptoms, and those with worse lung function early still had a faster rate of decline. Our findings suggest that predisposing genetic and/or environmental factors and events in infancy and preschool in children with CF may have a strong impact on lung function from early childhood through adolescence and adulthood, even in children diagnosed by newborn screening.

Our results are consistent with Burns’ finding that 97% of infants with CF in their clinic population had serologic or microbiologic evidence of *Pa* exposure before age three[43], which would suggest that growth of *Pa* on respiratory culture cannot be the driving force in differences in early lung function. In the GMS dataset, subjects with severe adolescent lung disease had mean early childhood FEV_1_ approximately 25 percentage points lower than adolescent subjects with mild disease, regardless of early childhood *Pa* status. In the NBS dataset, early lung function was lower in subjects with lower adolescent lung function. We acknowledge that there is an association between early *Pa* infection, early childhood lung function, and adolescent lung function, as subjects with lower adolescent lung function *and* early *Pa* infection appeared to have worse early childhood lung function in both study populations. This confirms that infection with *Pa* is one of many factors associated with lower lung function in adolescence, but our findings emphasize that, even when adjusting for chronicity or prevalence of positive cultures, *Pa* is not the *major* cause of lower lung function in children that persists into adolescence.

Previous studies have shown that lung function in healthy subjects remains relatively stable when comparing percent-predicted values from childhood to adolescence[44, 45]. Studies in children with CF have noted an association with higher baseline FEV_1_ and more rapid rate of decline over the next 3–6 years[27, 46]. However, we found that subjects with CF with lower adolescent lung function showed significantly greater rate of decline in FEV_1_ percent predicted *and* annual rate of decline in FEV_1_ percent predicted from early childhood to adolescence, while those subjects with higher adolescent lung function showed no significant decline from early childhood to adolescence. This suggests that children with CF and lower FEV_1_ are not simply tracking along a given lung function trajectory, but have active progression and worsening of their lung disease in comparison to their healthier counterparts with CF. Our findings may differ from previous studies in follow-up time from early childhood to adolescence was relatively longer–perhaps uniformly bridging adolescence in our population resulted in different patterns of lung function decline. The NBS population was also limited to a single state population, and thus our study population was smaller and possibly more homogenous. Finally, our analysis approaches may have differed.

The strongest predictor of low lung function in adolescence was low lung function in early childhood. Importantly, there were no significant differences in rate of decline when comparing *Pa* positive and negative subjects within adolescent lung function quartiles. Instead, subjects with lower adolescent lung function (in both *Pa* positive and negative groups) both started with lower early childhood lung function *and* showed a faster rate of decline in lung function than their counterparts with higher adolescent lung function. Those subjects with a higher percentage of respiratory cultures positive for *Pa* prior to age 6 also had lower lung function in adolescence, though that association was not as strong as that with early childhood lung function, and the dichotomous presence/absence of *Pa* prior to age 6 was not associated with adolescent lung function. This suggests that infectious burden, and possibly inflammatory response, plays a role in outcomes, though compliance with prescribed preventative treatments such as chest physiotherapy could also explain persistent decline in some children. Our findings are consistent with previous studies implicating chronic *Pa* infection in poorer pulmonary outcomes in patients with CF[15, 27, 29, 47], and implicating inflammation as a strong predictor of lung function decline[48].

The importance of *S*. *aureus* in CF lung disease has been a source of recent debate[28, 30, 31, 49, 50]; we found no significant association between *S*. *aureus* infection in early childhood and adolescent lung function, though we were unable to investigate the association with methicillin-resistant *S*. *aureus* due to low prevalence in our NBS population and lack of consistent reporting in our GMS population.

Poor nutritional status in early childhood was also associated with lower lung function in adolescence, which is consistent with previous studies[21, 22]. Earlier year of birth was also associated with poorer adolescent lung function, which may reflect our study design, as these subjects would have been older when adolescent lung function was recorded, but could also reflect advances in management and treatment. Our findings suggest early childhood lung function, early nutritional status, and density or persistence of early *Pa* infection were most strongly associated with adolescent lung function in subjects with CF.

Our two distinct datasets show consistent results, even though they represent different birth cohorts (straddling initiation of widespread use of inhaled tobramycin for treatment of *Pa* infection), had different genetic makeups and geographic origins, and likely reflected different treatment regimens (based on clinical advances from GMS to NBS birth cohorts). Both datasets had distinct advantages and complement the other well. The GMS dataset was large, representing CF centers across the U.S., and genetically homogenous (all F508del homozygotes), but was limited by use of annualized data, relatively scant data in infancy and preschool, and the extremes of phenotype design. The NBS dataset was smaller regional, but contained the full spectrum of CF lung disease (not extremes of phenotype), a representative distribution of CF genotypes, and used encounter-based, non-annualized data. Initiation of CF-directed care such as chest physiotherapy at diagnosis, and prior to development of respiratory symptoms, is another advantage in the NBS dataset and eliminates a potential confounder.

Our study had several limitations. Both GMS and NBS datasets are comprised of clinically collected data; there was not uniformity in data collection frequency or variables collected. PFT data at 6–8 years of age was the earliest age at which we could determine “early” childhood lung function. Because of the annualized data in the GMS study and the extremes of phenotype design, we were not able to explore associations between early childhood events, early childhood lung function, and adolescent lung function in great detail in this population. The NBS study population had more extensive early childhood data; however, there was still substantial variability as to how much data was available on individual subjects. We were unable to include mucoid *Pa* or methicillin-resistant *S*. *aureus* in either analysis due to low prevalence in the NBS population, and limited data available from the CFFPR for the majority of GMS subjects. Similarly, we had limited data on other organisms such as *Haemophilus influenza*, *Aspergillus fumigatus*, or *Stenotrophomonas maltophilia*, and were not able to include these in our analyses, nor were we able to include differences in therapies and treatments. Finally, socioeconomic status was not reliably collected and was therefore not included in our regression model, yet socioeconomic status has been shown to be associated with worse outcomes in CF[18]. Despite these limitations, we believe the consistency of our results in two distinct populations of subjects lend support to our findings.

In summary, we have shown in two distinct populations of subjects with CF that lung function in early childhood is more strongly associated with lung function in adolescence in CF than infection with *Pseudomonas aeruginosa* before age 6. Most strikingly, we found that subjects with lower lung function in adolescence had lower lung function in early childhood *and* a more rapid rate of decline of FEV_1_ from childhood to adolescence than those subjects with higher lung function in adolescence. Annual rate of decline was not different between *Pa* positive and *Pa* negative subjects in early childhood (within adolescent lung function quartile), suggesting that long-term pulmonary outcomes and disease trajectory may be largely determined prior to lung function measures at 6 years of age. Perhaps equally important, these differences were present in a cohort of children diagnosed with CF in infancy and prior to development of respiratory symptoms. Our findings suggest genetic influences and multiple factors in infancy and preschool have a substantial impact on lung function from early childhood onwards in subjects with CF. It will be critical to further study events in early childhood in patients with CF to define these key factors for future intervention.

## Supporting information

S1 Table GMS dataset. Variables for all subjects included in the final analysis of Gene Modifier Study population. Please contact Dr. Pittman with questions or for more details.
(XLSX)

S2 Table NBS dataset. Variables for all subjects included in the final analysis of the Newborn Screen Study population. Please contact Dr. Pittman with questions or for more details.
(XLSX)

## References

[1] Bush A. Six of the best: cystic fibrosis. Paediatr Respir Rev. 2001;2(4):287–93. Epub 2002/06/08. doi: 10.1053/prrv.2001.0162 12052299 10.1053/prrv.2001.0162

[2] Cutting GR, Zietlin P.L. Genetics and Pathophysiology of Cystic Fibrosis In: Chernick V, Boat T.F., Wilmott R.W., Bush A., editor. Kendig's Disorders of the Respiratory Tract in Children: Saunders Elsevier; 2006.

[3] Davies JC, Alton EW, Bush A. Cystic fibrosis. BMJ. 2007;335(7632):1255–9. Epub 2007/12/15. doi: 10.1136/bmj.39391.713229.AD 18079549 10.1136/bmj.39391.713229.AD PMC2137053

[4] Davis PB. Pulmonary Disease in Cystic Fibrosis In: Chernick V, Boat T.F., Wilmott R.W., Bush A., editor. Kendig's Disorders of the Respiratory Tract in Children. Philadelphia: Saunders Elsevier; 2006 p. 873–86.

[5] Moss RB. Infection, inflammation, and the downward spiral of cystic fibrosis lung disease. J Pediatr. 2009;154(2):162–3. Epub 2009/01/20. doi: 10.1016/j.jpeds.2008.09.042 19150671 10.1016/j.jpeds.2008.09.042

[6] Rosenfeld M, Gibson RL, McNamara S, Emerson J, Burns JL, Castile R, et al Early pulmonary infection, inflammation, and clinical outcomes in infants with cystic fibrosis. Pediatr Pulmonol. 2001;32(5):356–66. Epub 2001/10/12. 11596160 10.1002/ppul.1144

[7] Armstrong DS, Hook SM, Jamsen KM, Nixon GM, Carzino R, Carlin JB, et al Lower airway inflammation in infants with cystic fibrosis detected by newborn screening. Pediatr Pulmonol. 2005;40(6):500–10. Epub 2005/10/07. doi: 10.1002/ppul.20294 16208679 10.1002/ppul.20294

[8] Davis SD, Fordham LA, Brody AS, Noah TL, Retsch-Bogart GZ, Qaqish BF, et al Computed tomography reflects lower airway inflammation and tracks changes in early cystic fibrosis. Am J Respir Crit Care Med. 2007;175(9):943–50. Epub 2007/02/17. doi: 10.1164/rccm.200603-343OC 17303797 10.1164/rccm.200603-343OC

[9] Kozlowska WJ, Bush A, Wade A, Aurora P, Carr SB, Castle RA, et al Lung Function from Infancy to the Preschool Years Following Clinical Diagnosis of Cystic Fibrosis. Am J Respir Crit Care Med. 2008;178:42–9. Epub 2008/04/12. doi: 10.1164/rccm.200710-1599OC 18403721 10.1164/rccm.200710-1599OC

[10] Linnane BM, Hall GL, Nolan G, Brennan S, Stick SM, Sly PD, et al Lung Function in Infants with Cystic Fibrosis Diagnosed by Newborn Screening. Am J Respir Crit Care Med. 2008. Epub 2008/09/13. 10.1164/rccm.200804-551OC 18787217

[11] Lum S, Gustafsson P, Ljungberg H, Hulskamp G, Bush A, Carr SB, et al Early detection of cystic fibrosis lung disease: multiple-breath washout versus raised volume tests. Thorax. 2007;62(4):341–7. Epub 2006/11/24. doi: 10.1136/thx.2006.068262 17121870 10.1136/thx.2006.068262 PMC2092467

[12] Accurso FJ, Sontag MK. Gene modifiers in cystic fibrosis. J Clin Invest. 2008;118(3):839–41. Epub 2008/02/23. doi: 10.1172/JCI35138 18292812 10.1172/JCI35138 PMC2248430

[13] Corey M, Edwards L, Levison H, Knowles M. Longitudinal analysis of pulmonary function decline in patients with cystic fibrosis. J Pediatr. 1997;131(6):809–14. Epub 1998/01/15. 9427882 10.1016/s0022-3476(97)70025-8

[14] Davis PB. The decline and fall of pulmonary function in cystic fibrosis: new models, new lessons. J Pediatr. 1997;131(6):789–90. Epub 1998/01/15. 9427876 10.1016/s0022-3476(97)70019-2

[15] Emerson J, Rosenfeld M, McNamara S, Ramsey B, Gibson RL. Pseudomonas aeruginosa and other predictors of mortality and morbidity in young children with cystic fibrosis. Pediatr Pulmonol. 2002;34(2):91–100. Epub 2002/07/12. doi: 10.1002/ppul.10127 12112774 10.1002/ppul.10127

[16] Gan KH, Heijerman HG, Bakker W. Correlation between genotype and phenotype in patients with cystic fibrosis. N Engl J Med. 1994;330(12):865–6; author reply 6–7. Epub 1994/03/24. 8114850

[17] Schechter MS, Shelton BJ, Margolis PA, Fitzsimmons SC. The association of socioeconomic status with outcomes in cystic fibrosis patients in the United States. Am J Respir Crit Care Med. 2001;163(6):1331–7. Epub 2001/05/24. doi: 10.1164/ajrccm.163.6.9912100 11371397 10.1164/ajrccm.163.6.9912100

[18] Schechter MS. Non-genetic influences on cystic fibrosis lung disease: the role of sociodemographic characteristics, environmental exposures, and healthcare interventions. Semin Respir Crit Care Med. 2003;24(6):639–52. Epub 2005/08/10. doi: 10.1055/s-2004-815660 16088580 10.1055/s-2004-815660

[19] Collaco JM, Vanscoy L, Bremer L, McDougal K, Blackman SM, Bowers A, et al Interactions between secondhand smoke and genes that affect cystic fibrosis lung disease. JAMA. 2008;299(4):417–24. Epub 2008/01/31. doi: 10.1001/jama.299.4.417 18230779 10.1001/jama.299.4.417 PMC3139475

[20] Wolfenden LL, Schechter MS. Genetic and non-genetic determinants of outcomes in cystic fibrosis. Paediatr Respir Rev. 2009;10(1):32–6. Epub 2009/02/11. doi: 10.1016/j.prrv.2008.04.002 19203742 10.1016/j.prrv.2008.04.002

[21] McPhail GL, Acton JD, Fenchel MC, Amin RS, Seid M. Improvements in lung function outcomes in children with cystic fibrosis are associated with better nutrition, fewer chronic pseudomonas aeruginosa infections, and dornase alfa use. J Pediatr. 2008;153(6):752–7. Epub 2008/09/02. doi: 10.1016/j.jpeds.2008.07.011 18760423 10.1016/j.jpeds.2008.07.011

[22] Zemel BS, Jawad AF, FitzSimmons S, Stallings VA. Longitudinal relationship among growth, nutritional status, and pulmonary function in children with cystic fibrosis: analysis of the Cystic Fibrosis Foundation National CF Patient Registry. J Pediatr. 2000;137(3):374–80. Epub 2000/09/02. doi: 10.1067/mpd.2000.107891 10969263 10.1067/mpd.2000.107891

[23] Schaedel C, de Monestrol I, Hjelte L, Johannesson M, Kornfalt R, Lindblad A, et al Predictors of deterioration of lung function in cystic fibrosis. Pediatr Pulmonol. 2002;33(6):483–91. Epub 2002/05/10. doi: 10.1002/ppul.10100 12001283 10.1002/ppul.10100

[24] Rosenfeld M, Ramsey BW, Gibson RL. Pseudomonas acquisition in young patients with cystic fibrosis: pathophysiology, diagnosis, and management. Curr Opin Pulm Med. 2003;9(6):492–7. Epub 2003/10/10. 14534401 10.1097/00063198-200311000-00008

[25] Goss CH, Mayer-Hamblett N, Aitken ML, Rubenfeld GD, Ramsey BW. Association between Stenotrophomonas maltophilia and lung function in cystic fibrosis. Thorax. 2004;59(11):955–9. Epub 2004/11/02. doi: 10.1136/thx.2003.017707 15516471 10.1136/thx.2003.017707 PMC1746887

[26] Li Z, Kosorok MR, Farrell PM, Laxova A, West SE, Green CG, et al Longitudinal development of mucoid Pseudomonas aeruginosa infection and lung disease progression in children with cystic fibrosis. JAMA. 2005;293(5):581–8. Epub 2005/02/03. doi: 10.1001/jama.293.5.581 15687313 10.1001/jama.293.5.581

[27] Konstan MW, Morgan WJ, Butler SM, Pasta DJ, Craib ML, Silva SJ, et al Risk factors for rate of decline in forced expiratory volume in one second in children and adolescents with cystic fibrosis. J Pediatr. 2007;151(2):134–9, 9 e1. Epub 2007/07/24. doi: 10.1016/j.jpeds.2007.03.006 17643762 10.1016/j.jpeds.2007.03.006

[28] Sagel SD, Gibson RL, Emerson J, McNamara S, Burns JL, Wagener JS, et al Impact of Pseudomonas and Staphylococcus infection on inflammation and clinical status in young children with cystic fibrosis. J Pediatr. 2009;154(2):183–8. Epub 2008/09/30. doi: 10.1016/j.jpeds.2008.08.001 18822427 10.1016/j.jpeds.2008.08.001 PMC2654617

[29] Pittman JE, Calloway EH, Kiser M, Yeatts J, Davis SD, Drumm ML, et al Age of Pseudomonas aeruginosa acquisition and subsequent severity of cystic fibrosis lung disease. Pediatric Pulmonology. 2010. Epub 2011/01/05. 10.1002/ppul.21397 PMC4239995 21194167

[30] Dasenbrook EC, Merlo CA, Diener-West M, Lechtzin N, Boyle MP. Persistent methicillin-resistant Staphylococcus aureus and rate of FEV1 decline in cystic fibrosis. Am J Respir Crit Care Med. 2008;178(8):814–21. Epub 2008/08/02. doi: 10.1164/rccm.200802-327OC 18669817 10.1164/rccm.200802-327OC

[31] Levy H, Kalish LA, Cannon CL, Garcia KC, Gerard C, Goldmann D, et al Predictors of mucoid Pseudomonas colonization in cystic fibrosis patients. Pediatr Pulmonol. 2008;43(5):463–7 doi: 10.1002/ppul.20794 18361452 10.1002/ppul.20794 PMC3693457

[32] Waters V, Atenafu EG, Salazar JG, Lu A, Yau Y, Matukas L, et al Chronic Stenotrophomonas maltophilia infection and exacerbation outcomes in cystic fibrosis. Journal of cystic fibrosis: official journal of the European Cystic Fibrosis Society. 2012;11(1):8–13. Epub 2011/08/19. 21849265 10.1016/j.jcf.2011.07.008

[33] McKone EF, Emerson SS, Edwards KL, Aitken ML. Effect of genotype on phenotype and mortality in cystic fibrosis: a retrospective cohort study. Lancet. 2003;361(9370):1671–6. Epub 2003/05/28. doi: 10.1016/S0140-6736(03)13368-5 12767731 10.1016/S0140-6736(03)13368-5

[34] Braun AT, Farrell PM, Ferec C, Audrezet MP, Laxova A, Li Z, et al Cystic fibrosis mutations and genotype-pulmonary phenotype analysis. J Cyst Fibros. 2006;5(1):33–41. Epub 2005/11/09. doi: 10.1016/j.jcf.2005.09.008 16275171 10.1016/j.jcf.2005.09.008

[35] Drumm ML, Konstan MW, Schluchter MD, Handler A, Pace R, Zou F, et al Genetic modifiers of lung disease in cystic fibrosis. N Engl J Med. 2005;353(14):1443–53. Epub 2005/10/07. doi: 10.1056/NEJMoa051469 16207846 10.1056/NEJMoa051469

[36] Ramsey BW, Pepe MS, Quan JM, Otto KL, Montgomery AB, Williams-Warren J, et al Intermittent administration of inhaled tobramycin in patients with cystic fibrosis. Cystic Fibrosis Inhaled Tobramycin Study Group. N Engl J Med. 1999;340(1):23–30. Epub 1999/01/08. doi: 10.1056/NEJM199901073400104 9878641 10.1056/NEJM199901073400104

[37] Gibson RL, Emerson J, McNamara S, Burns JL, Rosenfeld M, Yunker A, et al Significant microbiological effect of inhaled tobramycin in young children with cystic fibrosis. Am J Respir Crit Care Med. 2003;167(6):841–9. Epub 2002/12/14. doi: 10.1164/rccm.200208-855OC 12480612 10.1164/rccm.200208-855OC

[38] Wang X, Dockery DW, Wypij D, Fay ME, Ferris BG Jr. Pulmonary function between 6 and 18 years of age. Pediatr Pulmonol. 1993;15(2):75–88. Epub 1993/02/01. 8474788 10.1002/ppul.1950150204

[39] Abman SH, Ogle JW, Harbeck RJ, Butler-Simon N, Hammond KB, Accurso FJ. Early bacteriologic, immunologic, and clinical courses of young infants with cystic fibrosis identified by neonatal screening. The Journal of pediatrics. 1991;119(2):211–7. Epub 1991/08/01. 1907318 10.1016/s0022-3476(05)80729-2

[40] Hammond KB, Abman SH, Sokol RJ, Accurso FJ. Efficacy of statewide neonatal screening for cystic fibrosis by assay of trypsinogen concentrations. The New England journal of medicine. 1991;325(11):769–74. Epub 1991/09/12. doi: 10.1056/NEJM199109123251104 1870650 10.1056/NEJM199109123251104

[41] Hankinson JL, Odencrantz JR, Fedan KB. Spirometric reference values from a sample of the general U.S. population. Am J Respir Crit Care Med. 1999;159(1):179–87. Epub 1999/01/05. doi: 10.1164/ajrccm.159.1.9712108 9872837 10.1164/ajrccm.159.1.9712108

[42] Kuczmarski RJ OC, Guo SS, et al CDC growth charts for the United States: Methods and development In: Statistics. NCfH, editor. Washington, DC: Vital Health Stat; 2000. 12043359

[43] Burns JL, Gibson RL, McNamara S, Yim D, Emerson J, Rosenfeld M, et al Longitudinal assessment of Pseudomonas aeruginosa in young children with cystic fibrosis. J Infect Dis. 2001;183(3):444–52. Epub 2001/01/03. doi: 10.1086/318075 11133376 10.1086/318075

[44] Stern DA, Morgan WJ, Wright AL, Guerra S, Martinez FD. Poor airway function in early infancy and lung function by age 22 years: a non-selective longitudinal cohort study. Lancet. 2007;370(9589):758–64. Epub 2007/09/04. PubMed Central PMCID: PMC2831283. doi: 10.1016/S0140-6736(07)61379-8 17765525 10.1016/S0140-6736(07)61379-8 PMC2831283

[45] Hibbert ME, Hudson IL, Lanigan A, Landau LI, Phelan PD. Tracking of lung function in healthy children and adolescents. Pediatric Pulmonology. 1990;8(3):172–7. Epub 1990/01/01. 2349010 10.1002/ppul.1950080308

[46] Cogen J, Emerson J, Sanders DB, Ren C, Schechter MS, Gibson RL, et al Risk factors for lung function decline in a large cohort of young cystic fibrosis patients. Pediatr Pulmonol. 2015;50(8):763–70. doi: 10.1002/ppul.23217 26061914 10.1002/ppul.23217 PMC5462119

[47] Kosorok MR, Zeng L, West SE, Rock MJ, Splaingard ML, Laxova A, et al Acceleration of lung disease in children with cystic fibrosis after Pseudomonas aeruginosa acquisition. Pediatr Pulmonol. 2001;32(4):277–87. Epub 2001/09/25. 11568988 10.1002/ppul.2009.abs

[48] Sagel SD, Wagner BD, Anthony MM, Emmett P, Zemanick ET. Sputum biomarkers of inflammation and lung function decline in children with cystic fibrosis. Am J Respir Crit Care Med. 2012;186(9):857–65. PubMed Central PMCID: PMCPMC3530222. doi: 10.1164/rccm.201203-0507OC 22904182 10.1164/rccm.201203-0507OC PMC3530222

[49] Rosenfeld M, Ramsey B. Evolution of airway microbiology in the infant with cystic fibrosis: role of nonpseudomonal and pseudomonal pathogens. Semin Respir Infect. 1992;7(3):158–67. Epub 1992/09/01. 1475540

[50] Sawicki GS, Rasouliyan L, Pasta DJ, Regelmann WE, Wagener JS, Waltz DA, et al The impact of incident methicillin resistant Staphylococcus aureus detection on pulmonary function in cystic fibrosis. Pediatr Pulmonol. 2008;43(11):1117–23. Epub 2008/10/11. doi: 10.1002/ppul.20914 18846559 10.1002/ppul.20914

